# Self-Reported Antidepressant Drug Side Effects, Medication Adherence, and Its Associated Factors among Patients Diagnosed with Depression at the Psychiatric Hospital of Nepal

**DOI:** 10.1155/2020/7024275

**Published:** 2020-10-17

**Authors:** Nirmal Raj Marasine, Sabina Sankhi, Rajendra Lamichhane, Nabin Raj Marasini, Nim Bahadur Dangi

**Affiliations:** ^1^Pharmaceutical Sciences Program, School of Health and Allied Sciences, Pokhara University, Pokhara-30, Kaski, Nepal; ^2^Department of Public Health, Asian College for Advance Studies, Purbanchal University, Lalitpur, Nepal; ^3^Department of Public Health, La Grande International College, Kaski, Nepal

## Abstract

**Objective:**

The present study is aimed at evaluating the side effects of antidepressant drugs, medication adherence (MA), and associated factors among patients diagnosed with depression at a psychiatric hospital in western Nepal.

**Methods:**

A prospective cross-sectional study was conducted among 174 patients visiting the outpatient clinic of a psychiatric hospital. The antidepressant side effect checklist (ASEC) was used to classify the reported antidepressant drug side effects into mild, moderate, and severe types. The Naranjo adverse drug reaction (ADR) probability scale was employed to assess the ADRs, and the Morisky Green Levine Adherence (MGLA) score was employed to determine the rate of medication adherence. Descriptive statistics and bivariate analysis were used, and a *P* value < 0.05 was taken as statistically significant in the multivariate analysis.

**Results:**

The patients were mostly female (55.74%), with a median (IQR) age of 32 (20) years. Approximately 74.13% of the patients experienced antidepressant side effects, where insomnia (17.05%) and anxiety (17.05%) were the most common. More than half of the patients (52.29%) had a low level of adherence. Females were 1.01 times more likely to be nonadherent to their antidepressant medications compared to males, adjusted odds ratio (AOR): 1.001 (0.31-1.63). Similarly, illiterate patients tended to be more nonadherent compared to literates, AOR: 1.342 (0. 93-2.82), and unemployed individuals were 1.5 times more likely to be nonadherent to their medications compared to employed individuals, AOR: 1.46 (1.16-4.13). Likewise, patients with severe side effects were more prone to develop nonadherence than those with moderate side effects, AOR: 1.173 (0.42-3.25). A significant association was found between the Naranjo score and medication adherence.

**Conclusions:**

This study suggests that antidepressant drug side effects were more prevalent and medication adherence was extremely poor among depressive patients in psychiatric hospitals. Factors such as gender, occupation, education, side effects, and ADRs attributed to poor medication adherence in patients.

## 1. Introduction

Depression, a common chronic psychiatric illness, interferes with physical and mental performance as well as the social life of the individual. More than 264 million people worldwide suffer from depression [1]. The World Health Organization (WHO) estimated that by year 2020, it would become the second-highest known cause of worldwide disability [2], and by 2030, it would possibly become the most significant factor contributing to the global burden of disease [3]. Nepal has a high prevalence of depression with the second-highest rate of “disability-adjusted life years” in the world [4]. In Nepal, 1 in every 3 people suffers from mental illness, and more than 90 percent of the population in need of mental health services has no access to treatment [4]. The first epidemiological field survey conducted in the Kathmandu valley by the government of Nepal in 1984 estimated the prevalence of mental illness to be approximately 14% [5].

Antidepressant medication is often considered to be the best treatment option for depression [6]. American Psychiatric Association (APA) guidelines (2010) has endorsed selective serotonin reuptake inhibitors (SSRIs), serotonin and noradrenaline reuptake inhibitors (SNRIs), and selective serotonin-noradrenaline reuptake inhibitors (SSNRIs) as first-line medication owing to their similar efficacy and lower overdose-related toxicity when compared to tricyclic antidepressants and monoamine oxidase inhibitors [7].

However, other factors, such as adverse effect profiles, cost, safety profile, history of prior medication treatment, and patient preference, are important in the initial selection of antidepressants and should be considered by healthcare professionals [7, 8]. In a study from the United States, it was reported that the patients with bothersome side effects are more likely to nonadhere to their antidepressant medications [9]. This affects the efficacy of antidepressant medications [10]. Moreover, antidepressant drug side effects are the significant determinants of antidepressant-associated nonadherence, which is predicted to be considerably high among depressive people [11]. The determination of drug-associated side effects will guide the development of policies for the effective management of severe and probable types of side effects, and medication adherence will contribute to the assessment of the effectiveness of interventions for better therapeutic outcomes. Therefore, this study is aimed at evaluating the antidepressant drug side effects, medication adherence (MA), and associated factors among patients diagnosed with depression at a psychiatric hospital in western Nepal.

## 2. Methods

### 2.1. Study Setting

A prospective cross-sectional study was conducted between August and October 2019, among patients visiting the outpatient department of B.G. Hospital. B.G. Hospital is a 25-bedded psychiatric hospital that provides both inpatient and outpatient services. It is the only psychiatric hospital in Kaski district, located in Pokhara-12, Kaski, Nepal. Easy accessibility makes it one of the busiest hospitals in Pokhara.

### 2.2. Sample Selection Criteria

All patients aged ≥18 years, diagnosed with depression, and under antidepressant medication for at least 1 month before the commencement of the study were included in the study. Pregnant or lactating mothers; those with a history of psychotic, bipolar disorder, or drug abuse; those with cognitive impairment; and those unable to communicate and understand the Nepali language were excluded from the study. To discard the patients with dementia, an upper age limit of 65 years was selected.

### 2.3. Sample Size

The calculated sample size for this study was 174. It was calculated based on the study conducted at two universities in Nepal in 2016 [12]. As per the study, the prevalence of depression was 11.7%. Setting the worst acceptable level at 5.0% and a confidence interval at 95.0%, a sample size of 158 was obtained using Epi Info 6. 10% of 158, i.e., 16, was added to make up for errors. Therefore, a total of 174 patients were enrolled in the study.

### 2.4. Study Variables

Antidepressant drug side effects and levels of medication adherence were our dependent variables. Likewise, sociodemographic characteristics of the patient, such as age, gender, education, occupation, and marital status, were the independent variables.

### 2.5. Data Collection Tools and Technique

Data were collected prospectively from the patients and their prescriptions by communicating with psychiatrists. Information on demographics (age, gender, education, marital status, religion, occupation, and residence) and antidepressants prescribed were collected. The antidepressant side effect checklist (ASEC), which was developed by the Royal College of the Psychiatrist, was employed to classify the reported side effects into mild, moderate, and severe types, as it consist of list of all the side effects associated with antidepressant drugs [13]. The Naranjo adverse drug reaction (ADR) probability scale, with 10 items, was employed to assess the antidepressants-associated ADRs. As per the scale, a score of >9 indicated definite ADR, 5-8 indicated probable ADR, 1-4 indicated possible ADR, and 0 indicated doubtful ADR [14]. Similarly, the Morisky Green Levine Adherence (MGLA) score was employed to determine the rate of medication adherence (MA). The MGLA score is a 4-item structured instrument in which four questions have dichotomous (yes, no) responses [15]. In this method, a low level of adherence was indicated by a score of 3 or 4, a moderate level of adherence was indicated by a score of 1 or 2, and a high level of adherence was indicated by a score of 0. Tools were translated in the Nepali language for easy understanding of the patients, and Cronbach's alpha of the tool was found to be 0.80. A clinical pharmacist collected the required data using a pretested structured data collection tool.

### 2.6. Data Collection Period

August to October 2019 was the data collection period for the study. Data were collected six days a week, i.e., from Sunday to Friday, at the outpatient department (OPD) between 11 a.m. and 4 p.m. (OPD timing).

### 2.7. Statistical Analysis

Data were entered in Microsoft Excel 2013. The entered data were transferred to SPSS version 20 for further analysis. Univariate, bivariate, and multivariate analysis were performed. In univariate analysis, the median, interquartile range, frequency, and percentage were calculated. Bivariate analysis of independent variables with the dependent/outcome variable was performed by cross-tabulation, and the Pearson chi-squared test was performed. Variables found significant in the bivariate analysis were included in a multivariate analysis and fitted using binary logistic regression (enter method) with nonadherence as an outcome variables. A measure of association was presented as an odds ratio (OR) with a 95.0% confidence interval (CI). *P* < 0.05 and *P* < 0.001 were considered statistically significant.

### 2.8. Ethics

Ethical approval and clearance for this study were obtained from the Institutional Review Committee (IRC) of Pokhara University Research Center (Ref.No.18-076-077), and data collection permission was taken from the hospital before data collection. Written informed consent was obtained from all patients before their involvement in the study, and all the informations were kept confidential.

## 3. Results

A total of 174 patients approached the study, giving a 100% response rate. More than half of the study population (97; 55.74%) was female with a median (IQR) age of 32 (20) years. The majority of thepatients (146; 83.9%) were Hinduism in religion. The study patients were predominantly married (120; 69%) and literate (124; 71.26%). Most of the patients were housewives (64; 36.78%) and resided in urban areas (133; 76.26%), as depicted in Table 1.

Out of 174 patients, 150 (86.20%) were on monotherapy. Of these, SSRIs were prescribed in 101 (58.04%) patients, SNRIs in 24 (13.79%), TCAs in 17 (9.77%), and atypical antidepressants in 8 (4.59%) patients, respectively. The most frequently prescribed antidepressant was fluoxetine (40; 22.98%), as shown in Table 2. Antidepressants prescribed as combination therapy are depicted in Table 3.

**Figure 1:**
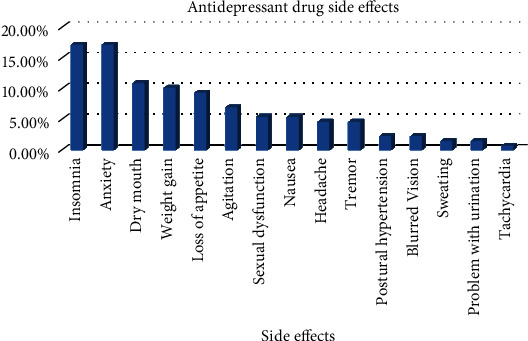
Frequency of patient-reported antidepressant drug side effects (*n* = 129).

Of approximately 129 (74.13%) patients encountered with antidepressant drugs side effects, the most common were insomnia and anxiety (22, 17.05%), followed by dry mouth (14; 10.85%) and weight gain (13; 10.07%), respectively. Tachycardia was rarely reported (1; 0.77%) by the patients. Patients reported antidepressant drug side effects are illustrated in Figure 1.

Based on ASEC, the majority of the reported side effects were moderate (98; 56.32%), followed by severe (22; 12.64%) and mild (9; 5.17%), respectively. Based on the Naranjo score, approximately 83.72% of ADRs were probable, and 16.27% were possible, as shown in Table 4.

Based on the scoring of MGLA, more than half of the patients (91; 52.29%) were found to have a low level of medication adherence. The average MGLA score obtained was 2.75 ± 1.42 out of 4, as depicted in Table 5.

Scores of medication adherence were used to categorize patients into a binary variable optimal and suboptimal. The association was considered statistically significant with an obtained “*P*” value of less than 0.05. According to the bivariate analysis, age, marital status, residence, religion, and prescribed antidepressants were not found to be correlated with the dependent variable. However, only four independent variables were found to be significantly associated with poor adherence, which are listed in Table 6. Females were 1.01 times more likely to be nonadherent to their antidepressant medications compared to males, adjusted odds ratio (AOR): 1.001 (0.31-1.63). Similarly, illiterate patients tended to be more nonadherent compared to literates, AOR: 1.342 (0. 93-2.82), and unemployed individuals were 1.5 times more likely to be nonadherent to their medications compared to employed individuals, AOR: 1.46 (1.16-4.13). Likewise, patients with severe side effects were more prone to develop nonadherence than those with moderate side effects, AOR: 1.173 (0.42-3.25). A significant association was found between the Naranjo score and medication adherence.

## 4. Discussion

This study evaluated the antidepressant drug side effects, medication adherence, and associated factors among depressive patients visiting the outpatient clinic in a psychiatric hospital in Nepal for three months among 174 patients. The majority of the patients were female, with a median (IQR) age of 32 (20) years. This study revealed that the antidepressant drug side effects were more prevalent and that the level of medication adherence was extremely poor among depressive patients. Although the findings represented a small number of patients, monotherapy was predominant in most of the patients, where SSRIs (fluoxetine, escitalopram, and sertraline) were the most frequently prescribed antidepressants, followed by SNRIs (duloxetine, venlafaxine) and TCA (amitriptyline), respectively. These findings were similar to several studies conducted globally. A study from Malaysia reported that SSRIs were the most frequently prescribed antidepressants whose prescriptions increased rapidly in the last decade [16]. Similar findings were also reported from the Europe [17]. A study from the United States revealed the increased prescription of SSRIs from 54.8% to 66.9% and declined prescription of TCAs from 35% to 11.1% between 1996 and 2005 [18]. The reason behind the frequent use of SSRIs compared to TCAs might be fewer side effects and better tolerability offered by SSRIs than the latter [19]. In contrast, amitriptyline (28.1%) followed by fluoxetine (27.2%) were the most often prescribed antidepressants as monotherapy in a study from Malaysia [11].

This study found that side effects are most common in depressive patients, as above 70% of the patients experienced antidepressant drug side effects. This finding was comparably higher than that of an Indian study (26.87%) [20] and lower than that of an Ethiopian study (85.7%) [10]. A total of 15 side effect types were identified in this study, where general ones such as insomnia, anxiety, dry mouth, and weight gain were more common among the study population. This finding was in contrast to that of a study conducted in India, wherein dry mouth was the most common, closely followed by nausea and tremor [21]. All side effects should be given equal attention, as a good number of them were moderate and probable as per the ASEC and Naranjo scales. Most of the reported side effects were not associated with discontinuation of antidepressants. However, some side effects might be strong predictors of antidepressant discontinuation. For example, sexual dysfunction, although relatively uncommon, could lead to nonadherence and poor improvement in mental condition, which can eventually increase suicidal ideation in patients [22]. As these side effects are treatable, specific attention is needed to enable collaborative decision-making and maximize the chance of an individual receiving effective medication with a minimal burden of adverse effects [23].

The study showed that the majority of patients with depression had a low level of adherence (52.29%) towards antidepressant medications. This finding showed consistency with two other studies conducted in Ethiopia and Saudi Arabia, both of which ascertained lower adherence to antidepressant medications, i.e., 52.29% and 57.1%, respectively [10, 24]. In this study, females were found more likely to be nonadherent than males with AOR: 1.001 (0.31-1.63). In Nepal, women play multiple roles in the family and society, such as homemakers, spouses, mothers, professionals, and caregivers, which might cause them difficulty in making time for hospital visits, making them nonadherent to their prescribed medications. A similar study from Canada also reported on men with better compliance with their antidepressant medication than women [25]. However, a finding opposite to this study was reported from the USA and Belgium, wherein men were found more likely to discontinue their treatment even without consent from their physicians [26, 27]. This study showed unemployment, the reason for medication nonadherence. This is because such patients cannot afford medicines for the long term, and frequent appointments with their physicians become expensive for them. Medication adherence was found to be significantly associated with the probability and severity of antidepressant drug adverse effects/side effects at a “*P*” value of 0.05. This contradicts the finding of the Ethiopian study [10]. In this study, illiterate patients were observed to be more nonadherent than literate patients. This might be due to illiterate patients being afraid of the drug side effects owing to their limited knowledge about diseases and drugs, in addition to their unwillingness to discuss the same with medical professionals. Similar findings regarding poor compliance among less-educated patients were reported in a study from Spain [28].

## 5. Limitation and Strength of the Study

There are some limitations to this study. It was limited to a single center in western Nepal, which along with its small sample size shrinks the power of the study to determine the factors associated with antidepressants adherence. A self-reported medication adherence tool was used, which might be associated with subjective bias. Hence, other experimental methods, such as pill count and mechanical device recording, could strengthen the findings on medication adherence. Additionally, patients who were unable to speak and understand the Nepali language were discarded, which might be associated with language bias. However, the findings of this study are expected to have a good impact on the education of psychopharmacology.

## 6. Conclusions

This study revealed that in depressive patients, self-reported antidepressant drugs side effects were highly prevalent, and anxiety, insomnia, dry mouth, and weight gain were more frequent. Medication adherence was extremely poor in a psychiatric hospital. This was attributed to factors such as gender, occupation, education, side effects, and ADRs. Psychiatrists should carry out adherence-enhancing interventions for their patients. Education and assurance of patients about side effects associated with medications will enhance their adherence to antidepressants and hence improves the therapeutic outcomes.

## Figures and Tables

**Table 1:** Sociodemographic characteristics of patients (*n* = 174).

Characteristics	Category	*n* (%)
Gender	Female	97 (55.74)
	Male	77 (44.25)
Education	Literate	124 (71.26)
	Illiterate	50 (28.73)
Marital status	Married	120 (69.0)
	Single	54 (31.0)
Religion	Hinduism	146 (83.9)
	Buddhism	12 (6.9)
	Christianity	6 (3.4)
	Muslim	10 (5.7)
Occupation	Business	24 (13.8)
	Employment	57 (32.8)
	Farmer	21 (12.06)
	Housewife	64 (36.78)
	Others	8 (4.59)
Residence	Urban	133 (76.43)
	Rural	41 (23.56)
^†^Age	18 to 65	32 (20)

^†^Median (IQR) instead of *n* (%).

**Table 2:** Antidepressants prescribed as monotherapy (*n* = 174).

Class of drugs	*n* (%)	Individual drugs, *n* (%)
Selective serotonin reuptake inhibitor (SSRIs)	101 (58.04)	Fluoxetine (40; 22.98) Sertraline (24; 13.79) Escitalopram (37; 21.12)
Serotonin and noradrenaline reuptake inhibitors (SNRIs)	24 (13.79)	Duloxetine (15; 8.62) Venlafaxine (9; 5.17)
Tricyclic antidepressants	17 (9.77)	Amitriptyline (17; 9.77)
Atypical antidepressants	8 (4.59)	Bupropion (3; 1.72) Mirtazapine (5; 2.87)

**Table 3:** Antidepressants prescribed as a combination therapy (*n* = 174).

Drugs	*n* (%)
Fluoxetine+bupropion	8 (4.59)
Sertaline+mirtazapine	9 (5.17)
Sertraline+amitriptyline	4 (2.29)
Fluoxetine+amitriptyline	3 (1.72)

**Table 4:** Severity of side effects and probability of ADRs (*n* = 129).

Variables		*n* (%)
Severity	Mild	9 (5.17)
	Moderate	98 (56.32)
	Severe	22 (12.64)
Probability	Probable	108 (83.72)
	Possible	21 (16.27)

**Table 5:** Level of medication adherence (*n* = 174).

Level of adherence	*n* (%)
Low	91 (52.29)
Moderate	53 (30.45)
High	30 (17.24)

**Table 6:** Factors associated with medication adherence.

Variables	Level of adherence		COR (95% CI)	AOR (95% CI)
	Optimal	Suboptimal		
*Sex*				
Male	24	53	1	1
Female	30	67	1.011 (0.51-1.83)^∗^	1.001 (0.31-1.63)^∗∗^
*Education*				
Literate	46	78	1	1
Illiterate	9	41	2.686 (1.09-3.88)^∗^	1.342 (0.93-2.82)^∗∗^
*Occupation*				
Employed	27	30	1	1
Unemployed	28	89	2.86 (1.29-3.02)^∗∗^	1.46 (1.16-4.13)^∗^
*Naranjo score*				
Possible	12	9	1	1
Probable	30	78	3.46 (1.27-6.18)^∗∗^	2.738 (0.92-5.70)^∗∗^
*Severity of ADR*				
Moderate	48	60	1	1
Severe	5	16	2.56 (0.96-5.31)^∗^	1.173 (0.42-3.25)^∗∗^

^∗^Significant at *P* < 0.05. ^∗∗^Significant at *P* < 0.001. Abbreviation: COR: crude odds ratio; AOR: adjusted odds ratio; ADR: adverse drug reaction.

## Data Availability

The raw data used to support the findings of this study are made available from the corresponding author upon reasonable request.

## Supplementary Materials

Supplementary Materials The supplementary file contains the data collection sheet.
