# Supplementary material for: Self-Reported Antidepressant Drug Side Effects, Medication Adherence, and Its Associated Factors among Patients Diagnosed with Depression at the Psychiatric Hospital of Nepal
Source: Depress Res Treat. 2020 Oct 17;2020:7024275. doi: 10.1155/2020/7024275 (PMC7593732; doi:10.1155/2020/7024275)
Supplement: Supplementary Materials — The supplementary file contains the data collection sheet. [file 7024275.f1.docx]

**Data collection sheet**

**PERSONAL RECORD**

**Patient no:**………………………

**Age:** ………………………………………. **Address:**……………………………

**Contact no:**…………………………

**Residence:**

**Gender:
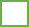
** male
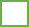
 female

**Education:
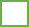
** literate
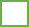
 illiterate

**Marital status:
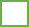
** married
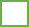
 unmarried
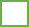
 divorced

**Religion:
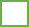
** Hinduism
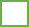
 Buddhism
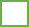
 Christianity
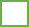
 Muslim
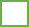
 Others **Occupation:
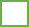
** Business
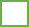
 Service
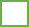
Agriculture
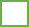
Housewife
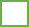
Others **Family history of depression:
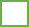
** Yes
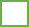
 No

**What is the reason for your depression?**


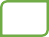
 Family history
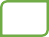
 Loneliness
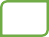
 Stressful events
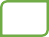
 Illness
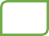
 Alcohol and drugs
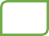
 Pregnancy

Others: ………………….

**Clinical characteristics of depression**


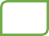
 Persistent sadness or low mood
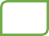
 Marked loss of interests or pleasure
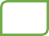
 Loss of energy
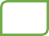
 Disturbances with sleep


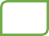
 Changes in appetite
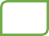
 Depressed mood


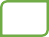
 Feelings of worthlessness
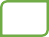
 Focusing on the negative aspects of the situation


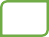
 Diminished ability to concentrate and difficulties with attention


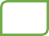
 Thoughts of self-harm
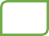
 Death or suicide others…

**Severity of depression**

- Sub threshold depressive
- Mild
- Moderate
- Severe

**Anti-depressant used and dose:** …………………………………………………….

**Antidepressants Side Effects Checklist (ASEC)**

Please score the following list of symptoms 0 = absent, 1 = mild, 2 = moderate, 3 = severe

| **S.N** | **ADR** | **0** | **1** | **2** | **3** | **Comment** |
| --- | --- | --- | --- | --- | --- | --- |
| 1 | Dry Mouth |  |  |  |  |  |
| 2 | Drowsiness |  |  |  |  |  |
| 3 | Insomnia |  |  |  |  |  |
| 4 | Blurred vision |  |  |  |  |  |
| 5 | Headache |  |  |  |  |  |
| 6 | Constipation |  |  |  |  |  |
| 7 | Diarrhea |  |  |  |  |  |
| 8 | Increased appetite |  |  |  |  |  |
| 9 | Decreased appetite |  |  |  |  |  |
| 10 | Nausea or vomiting |  |  |  |  |  |
| 11 | Problem with urination |  |  |  |  |  |
| 12 | Problem with sexual function |  |  |  |  |  |
| 13 | Palpitation |  |  |  |  |  |
| 14 | Feeling light- headed on standing |  |  |  |  |  |
| 15 | Feeling like room is spinning |  |  |  |  |  |
| 16 | Sweating |  |  |  |  |  |
| 17 | Increased body temperature |  |  |  |  |  |
| 18 | Tremor |  |  |  |  |  |
| 19 | Disorientation |  |  |  |  |  |
| 20 | Yawning |  |  |  |  |  |
| 21 | Weight gain |  |  |  |  |  |

Q1. What other symptoms have you had since the antidepressant medication (or since last completing the ASEC) that you think may be side-effects of the medication?

………………………………………………………………………………………………………….

**Naranjo Algorithm - ADR Probability Scale**

| S.N | Questions | Yes | No | Do not know | | Score | |
| --- | --- | --- | --- | --- | --- | --- | --- |
| 1 | Are there previous conclusive reports on this reaction? | +1 | 0 | 0 | |  | |
| 2 | Did the adverse event appear after the suspected drug  was administered? | +2 | -1 | 0 | |  | |
| 3 | Did the adverse event improve when the drug was discontinued or a specific antagonist was administered? | +1 | 0 | 0 | |  | |
| 4 | Did the adverse event reappear when the drug was re- administered? | +2 | -1 | 0 | |  | |
| 5 | Are there alternative causes that could on their own have caused the reaction? | 1 | +2 | 0 | |  | |
| 6 | Did the reaction reappear when a placebo was given? | -1 | +1 | 0 | |  | |
| 7 | Was the drug detected in blood or other fluids in concentrations known to be toxic? | +1 | 0 | 0 | |  | |
| 8 | Was the reaction more severe when the dose was increased or less severe when the dose was decreased? | +1 | 0 | 0 | |  | |
| 9 | Did the patient have a similar reaction to the same or similar drugs in any previous exposure? | +1 | 0 | 0 | |  | |
| 10 | Was the adverse event confirmed by any objective evidence? | +1 | 0 | 0 | |  | |
| Total= | | | | |  | |  |

Score of >9 indicated definite ADR, 5-8 indicated probable ADR, 1-4 indicated possible ADR, and 0 indicated doubtful ADR

**Morisky Green Levine Adherence score**

| **MGLA questions** | **Yes** | **No** |
| --- | --- | --- |
| Ever not taking medicine due to forgetfulness |  |  |
| Doing negligence about taking medicine |  |  |
| Stop medicine sometimes when feeling better |  |  |
| Stop taking medicine when feeling worse |  |  |
